# Aβ42 treatment of the brain side reduced the level of flotillin from endothelial cells on the blood side via FGF-2 signaling in a blood–brain barrier model

**DOI:** 10.1186/s13041-023-01005-1

**Published:** 2023-01-26

**Authors:** Tomohisa Nakamura, Tadahiro Hashita, Yuxin Chen, Yuan Gao, Yang Sun, Sadequl Islam, Hiroyuki Sato, Yasuyuki Shibuya, Kun Zou, Tamihide Matsunaga, Makoto Michikawa

**Affiliations:** 1 Department of Biochemistry, Graduate School of Medical Sciences, Nagoya City University, Kawasumi 1, Mizuho-Cyo, Mizuho-Ku, Nagoya, 467-8601 Japan; 2 Department of Clinical Pharmacy, Graduate School of Pharmaceutical Sciences, Nagoya City University, 3-1 Tanabe-Dori, Mizuho-Ku, Nagoya, 467-8603 Japan; 3 Department of Maxillofacial Surgery, Graduate School of Medical Sciences, Nagoya City University, Kawasumi 1, Mizuho-Cyo, Mizuho-Ku, Nagoya, 467-8601 Japan

**Keywords:** Amyloid β-protein, Alzheimer’s disease, Blood flotillin, Microvascular endothelial cells

## Abstract

Our previous study showed that the flotillin level is decreased in the blood of patients with Alzheimer’s disease (AD) when compared to that of patients with non-AD and vascular dementia; however, the molecular mechanism remains to be determined. In this study, to elucidate whether Aβ accumulation in the brain has an effect on the blood flotillin level, we used our previously established blood–brain barrier (BBB) culture model using microvascular endothelial cells obtained from human induced pluripotent stem cells (iBMECs) and astrocytes prepared from rat cortex. In this BBB model with iBMECs plated on the upper compartment (blood side) and astrocytes plated on the lower compartment (brain side), the trans-endothelial electrical resistance values are high (over 1500 Ωm^2^) and stable during experiments. We found that the addition of Aβ42 (0.5 and 2 µM) to the brain side significantly reduced the level of flotillin secreted by iBMECs on the blood side. The level of basic fibroblast growth factor (FGF-2) in the brain side was significantly reduced by Aβ42 treatment, and was accompanied by a reduction in the level of phosphorylation of the fibroblast growth factor receptor in iBMECs. The brain-side Aβ42 treatment-induced reduction of flotillin secretion into the blood side was restored in a dose-dependent manner by the addition of FGF-2 into the brain side. These results indicated that Aβ accumulation in the brain side reduced FGF-2 release from astrocytes, which attenuated FGF-2-mediated iBMECs signaling via the FGF-2 receptor, and thereby reduced flotillin secretion from iBMECs on the blood side. Our findings revealed a novel signaling pathway crossing the BBB from the brain side to the blood side, which is different from the classical intramural periarterial drainage or lymphatic-system-to-blood pathway.

**Supplementary Information:**

The online version contains supplementary material available at 10.1186/s13041-023-01005-1.

## Introduction

Alzheimer’s disease (AD) is a progressive neurodegenerative disease that is the most common type of dementia. It is clinically characterized by the loss of cognitive function accompanied by the deposition of extracellular amyloid β-protein (Aβ) aggregates called amyloid plaques, and the formation of intracellular neurofibrillary tangles that consist of phosphorylated tau [1, 2].

The diagnosis of AD is made on a clinicopathological basis, and a definitive diagnosis cannot be made until post-mortem confirmation of the presence of Aβ plaques and neurofibrillary tangles [3]. Therefore, it is difficult to make a differential diagnosis of AD, and to accurately predict the prognosis and monitor the disease based on clinical evidence alone, even for dementia specialists [2]. It is well-known that markers of Aβ and tau protein in the cerebrospinal fluid and brain Aβ deposition on positron-emission tomography (PET) are the most promising diagnostic markers [4]. However, cerebrospinal fluid and PET examinations are very invasive and/or expensive, and their clinical use is limited to only a few specialized centers.

To facilitate the diagnosis of AD, many studies have been performed to identify markers, including blood-based biomarkers, for clinical diagnosis [2, 4]. In addition, promising results have been reported from clinical trials of disease-modifying drug candidates, mainly immunotherapies targeting the Aβ pathology [2, 5]; thus the use of biomarkers has become more important than ever in terms of ensuring that AD patients have access to the appropriate therapies available and for an accurate prognosis in the early stages of their disease.

We recently showed that flotillin may serve as a blood marker for the estimation of brain amyloid deposition and early diagnosis of AD [6]. Flotillin-1 and -2 (reggie-2 and -1) were first discovered as proteins expressed by retinal ganglia during axon regeneration [7], and have since been widely used as markers for lipid rafts and exosomes. Flotillin-1 and -2 are ubiquitously expressed and highly conserved proteins that are involved in multifarious cellular events, including cell signaling, endocytosis, protein trafficking, and gene expression. However, the molecular mechanism by which the level of flotillin in blood decreases in patients with AD remains unknown.

In AD, abnormal accumulation of Aβ, tau phosphorylation, and inflammation occur in the brain, and it is assumed that Aβ accumulation in the brain has an effect on endothelial cells facing the brain side and also facing the blood side, and that it leads to reduced flotillin secretion from endothelial cells on the blood side. In this study, we examined the effect of Aβ treatment of the brain side on flotillin release from endothelial cells on the blood side using a BBB co-culture system (in vitro BBB model) [8].

## Material and methods

### Astrocyte culture

Primary astrocyte cultures were prepared from new born Wistar-rat (Japan SLC, Inc., Shizuoka, Japan) as described previously [9]. In brief, brains of postnatal day 1 rat were minced and incubated in Dulbecco’s modified Eagle’s medium (DMEM) in the presence of 100 µg/mL DNase I and 0.25% trypsin. Dissociated cells were plated on T75 flasks. After 10 days of incubation, flasks were shaken for 1 h at 37 °C to eliminate oligodendrocyte. The enriched astrocyte was checked by immunostaining for glial fibrillary acidic protein (GFAP), and re-plated onto different cell culture substrates. The culture of astrocyte that had been in vitro for less than 4 weeks were used throughout.

### Differentiation of human induced pluripotent stem (iPS) cells into iBMELCs

Brain microvascular endothelial-like cells (iBMECs) were prepared as previously described [8]. In Brief, human iPS cells were cultured in iPSC medium (Dulbecco’s Modified Eagle’s Medium/Ham’s F12 (Wako Pure Chemical Industries (Wako), Osaka, Japan) containing 20% knockout serum replacement (Invitrogen, Carlsbad, CA, USA), 2 mM l-glutamine (Wako), 1% minimal essential medium with non-essential amino acids (Invitrogen), and 0.1 mM β-mercaptoethanol (Sigma-Aldrich, St. Louis, MO, USA)) at 37℃ in 5% CO_2_ for 6 days. Then the culture medium was changed to EC medium (Human Endothelial-SFM (Thermo Fisher Scientific, Waltham, MA, USA) supplemented with 1% platelet-poor plasma derived bovine serum (PDS) (Alfa Aesar, Haverhill, MA, USA), 20 ng/mL FGF2, and 10 µM all-*trans* retinoic acid (RA) (Tocris Bioscience, Bristol, UK)). After 2 days, the cell were detached by Accutase (Nacalai Tesque, Kyoto, Japan) and re-prated on 0.3-cm^2^ Transwelll-Clear permeable inserts (0.4 µm pore size, Sabeu GmbH & CO. KG, Northeim, Germany) coated with a mixture of fibronectin (100 µg/mL; Wako) and collagen IV (400 µg/mL; Nitta gelatin, Osaka, Japan). These cells were then used to construct the in vitro BBB model.

### In vitro blood–brain barrier (BBB) model

**Fig. 1:**
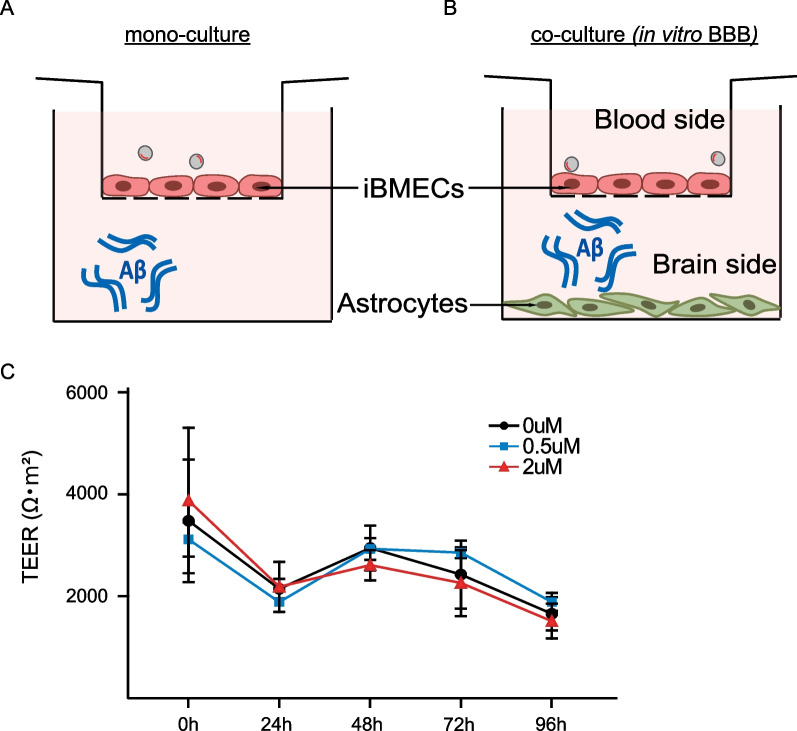
**A**, **B** Schematic diagram of the in vitro BBB model comprising brain endothelial cells and astrocytes. In the in vitro BBB model, an insert membrane was used to construct the BBB, and brain endothelial cells were seeded onto the upper surface of the insert membrane, and astrocytes were seeded on the bottom of the wells. The upper side was taken to be the “blood side”, and the bottom side was taken to be the “brain side”. **C** Effects of Aβ on the TEER values in the in vitro BBB model. Statistical significance was calculated using one-way analysis of variance (ANOVA) and the Tukey test

To construct in vitro models of BBB, iBMECs (3.0 × 10^5^ cells/insert) seeded on the upper side of the Transwell inserts were placed in the well of 24-well culture plates containing no cells (iBMECs mono-culture). Astrocyte were plated on the 24-well plates with DMEM containing 10% FBS and 1% penicillin/streptomycin until the experiment. Finally, astrocytes were replaced with serum-free medium before the experiment. Under these conditions, in vitro BBB models were constructed as shown on the scheme (Fig. 1A). For the experiment to examine the effect of Aβ42 in the brain on BBB, Aβ42 was added to the medium of the basal side of the 24-well culture plates.

### Trans-endothelial electrical resistance (TEER) measurement

Barrier integrity in in vitro BBB models were analyzed by measurement of TEER.

TEER values were measured using Millicell-ERS (Merck Millipore, Burlington, MA, USA) according to the manufacturer’s instructions. During the measurement of the time-course of TEER values, the medium was not changed.

### Western blot analysis

The conditioned medium were collected, and the cultured cells were lysed with RIPA buffer (50 mM Tris–HCl, pH 7.6, 150 mM NaCl, 1% NP-40, 0.5% sodium deoxycholate, 0.1% SDS) containing a protease inhibitor cocktail (Roche, Mannheim, Germany) and the phosphatase inhibitor cocktail solution I (FUJIFILM Wako Pure Chemical Corporation, Tokyo, Japan). Protein concentration were determined a BCA protein assay kit (Thermo Fisher Scientific, IL, USA). The protein samples were separated by SDS-PAGE and transferred to polyvinylidene difluoride membranes (Millipore, Billerica, MA, USA), which were blocked with 5% skim milk in TBS-T, and incubated overnight at 4℃ with the following primary antibodies: Flotillin-1 (Product No, sc-133153, Santa Cruz Biotechnology, CA, USA), Flotillin-2 (BD Transduction Laboratories), FGF-2 (S Santa Cruz Biotechnology, CA, USA). Analysis of protein was carried out using HRP-conjugated secondary antibodies, and chemiluminescence signals were detected using Amersham Imager 680 (GE Healthcare, MA, USA).

### ELISA

Prior to ELISA analysis, the CMs were concentrated up to about tenfold by ultrafiltration (Vivaspin500, SARTORIUS, Gloucestershire, UK). Each CM was then adjusted to a 4-fole concentration and use in the experiment. FGF-2 levels were determined using FGF-2 ELISA kit (Quantikine®ELISA, R&D systems, MN, USA), which is a quantitative sandwich enzyme immunoassay technique, according to the manufacturer’s instructions.

### Statistical analyses

Statistical analyses were performed using GraphPad prism 7.0 software (CA, USA). All values are presented as the mean ± S.E. of at least three independent experiments. Student's *t* test was used to determine whether the results were significantly different between two groups. We compared group difference with one-way analysis of variance (ANOVA) followed by Tukey's multiple-comparison test for two or more groups against a control group. A *p* value of < 0.05 was considered to represent a significant difference.

## Results

### Aβ42 decreases flotillin secretion from iBMECs in the in vitro BBB model

First, to determine whether Aβ42 affects flotillin secretion from brain microvascular endothelial cells (BMECs), we used a mono-layer culture model using iPS-derived BMECs (iBMECs) prepared from human iPS cells as reported previously [8] (Fig. 1A). Western blot analysis was performed using apical conditioned medium (CM) collected from iBMECs cultured for 96 h in serum-free media on the upper side (blood side) of the double chamber. We found that Aβ42 treatment of the brain side did not affect flotillin secretion from iBMECs on the blood side (Additional file 1: Fig. S1A, B).

**Fig. 2:**
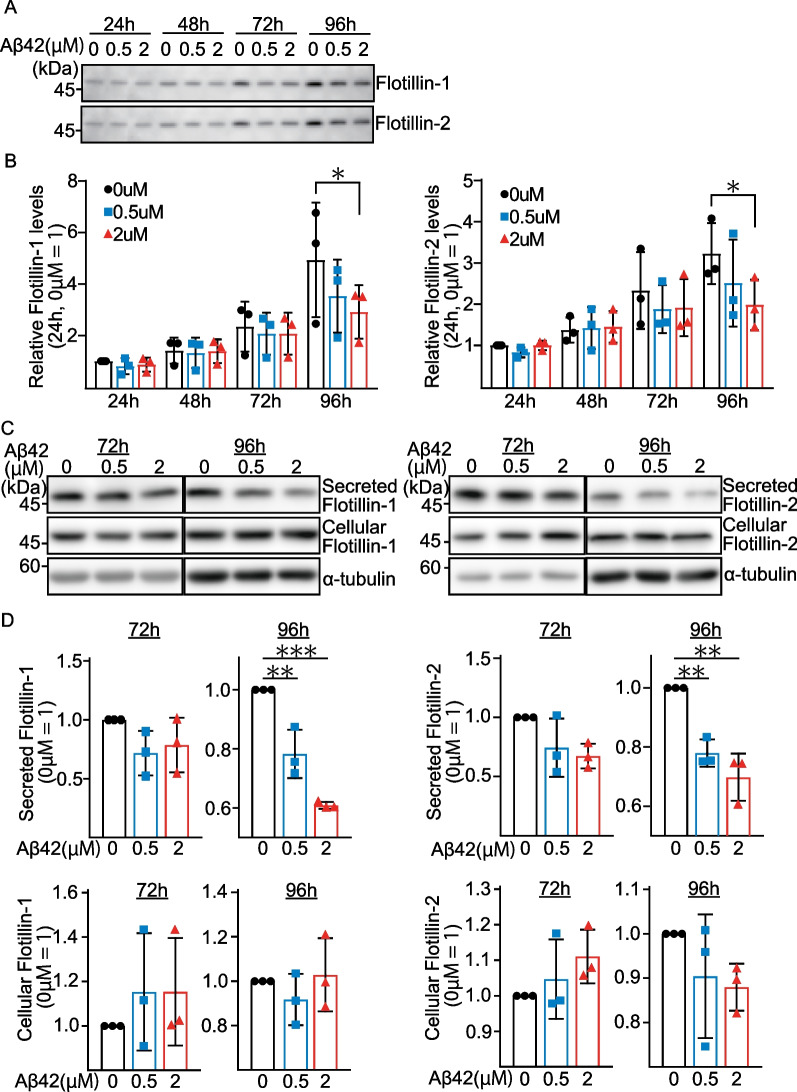
Aβ42 treatment decreased the flotillin secretion level in the blood-side CM, but did not alter the cellular flotillin level. **A** The level of flotillin secretion in the blood side increased in a time-dependent manner. The levels of flotillin-1 and -2 in the CM were analyzed by western blotting. **B** The levels of flotillin-1 and -2 secretion in the blood side were decreased by Aβ treatment, as determined using a fluorogenic substrate. Statistical significance was calculated using one-way ANOVA and the Tukey test (*p < 0.05). Data are shown as the mean ± standard deviation (SD; *n* = 3). **C** Cellular flotillin levels were determined by western blotting and densitometry. **D** Quantification of the protein levels of flotillin-1 and -2 normalized to α-tubulin, and expressed as values relative to the control. Statistical significance was calculated using one-way ANOVA and the Tukey test (*p < 0.05, **p < 0.01). Data are shown as the mean ± SD (*n* = 3)

It is well-known that astrocytes secrete a variety of factors that contribute to the maintenance of the functions of BMECs in the BBB [10]. In light of this, we next examined the effect of Aβ42 in the in vitro BBB model (Fig. 1B). In this culture model, the high trans-endothelial electrical resistance (TEER) values were over 1,500 Ωm^2^ (Fig. 1C) during the experiments and the values are much higher than those obtained with other models using rodent cells [11, 12]. In this culture, Aβ42 was added into the lower chamber of the double-floored culture system (Fig. 1B), and the cells were cultured for another 24, 48, 72, and 96 h. CM samples from each time point were collected and stored at -80 °C until use. Western blot analysis revealed that the flotillin-1 and -2 levels in the blood-side CM increased in a time-dependent manner, and that they were suppressed by Aβ42 treatment; the levels were significantly lower after Aβ42 treatment for 96 h (Flot-1: *p* = 0.045, Flot-2: *p* = 0.043) (Fig. 2A). As shown in Fig. 2B, the levels of flotillin-1 and -2 in the blood-side CM were significantly lower in the CM with the 2 µM Aβ42 treatment than in the control CM at 96 h. On the other hand, we found that Aβ42 did not alter the cellular flotillin-1 and -2 levels (Fig. 2C, D). Interestingly, Aβ42 did not have any effect on flotillin secretion into the blood side when iBMECs were cultured without astrocytes (Additional file 1: Fig. S1A, B). These results suggested that although Aβ does not have a direct effect on iBMECs to regulate flotillin release from iBMECs, it has an indirect effect via astrocytes, from which some factor(s) may be released to regulate flotillin secretion from iBMECs into the blood-side CM.

### Decreased FGF-2 production and secretion in astrocytes of the in vitro BBB model

**Fig. 3:**
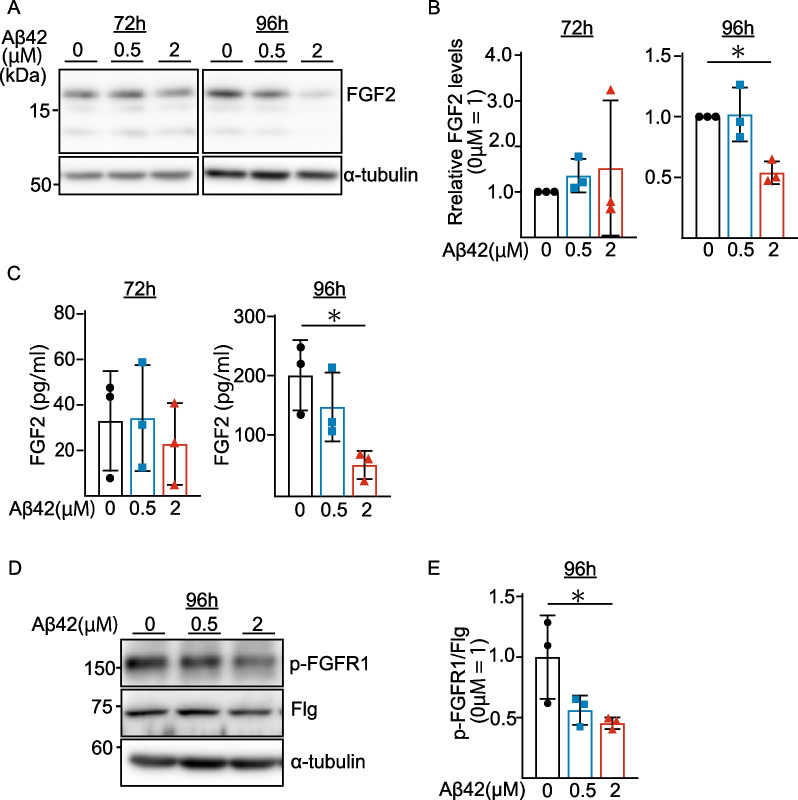
The cellular and secreted FGF-2 levels were decreased in astrocytes when Aβ42 was added into the brain side. **A** Western blot analysis of intracellular FGF-2. **B** Quantification of the FGF-2 protein levels normalized to α-tubulin, and expressed as values relative to the control. Statistical significance was calculated using one-way ANOVA and the Tukey test (*p < 0.05). Data are shown as the mean ± SD (*n* = 3). **C** Secreted FGF-2 in the brain side was measured using FGF-2 ELISA kits. Statistical significance was calculated using one-way ANOVA and the Tukey test (*p < 0.05). Data are shown as the mean ± SD (*n* = 3). The Aβ42 treatment-induced decrease in the FGF-2 levels regulated the phosphorylation levels of FGFR1 in iBMECs. **D** Protein levels were determined by western blotting and quantified by densitometry. **E** Quantification of phosphorylated (p-) FGFR1 levels normalized to Flg/FGFR1 levels, and expressed as values relative to the control. Statistical significance was calculated using one-way ANOVA and the Tukey test (**p < 0.01). Data are shown as the mean ± SD (*n* = 3)

To identify the factor(s) secreted by astrocytes, that have an effect on iBMECs to regulate flotillin release into the upper chamber (blood side), we used the in vitro BBB model shown in Fig. 1B. Previous studies have shown that basic fibroblast growth factor (FGF-2) released from astrocytes affects the function of the BBB [13, 14]. We examined whether the FGF-2 levels are associated with Aβ42 treatment in the in vitro BBB model. As shown in Fig. 3, we found that the cellular FGF-2 level was significantly reduced in astrocytes when Aβ42 was added into the lower camber (brain side) at a concentration of 2 µM for 96 h (Fig. 3A, B). We next measured the FGF-2 level in the brain-side CM by enzyme-linked immunosorbent assay (ELISA). As shown in Fig. 3C, Aβ42 treatment significantly decreased the FGF-2 level in the brain-side CM. In contrast, Aβ42 did not affect the FGF-2 level in the in vitro BBB models using iBMECs or astrocytes alone (Additional file 1: Figs. S2A, B, 3A, B). We also found that co-culture of iBMECs and astrocytes significantly increased FGF-2 levels in astrocytes (Additional file 1: Fig. S4A, B), suggesting that the iBMECs release factors to increase FGF-2 levels in astrocytes.

### FGF-2 restored the Aβ42-induced decrease in flotillin secretion

**Fig. 4:**
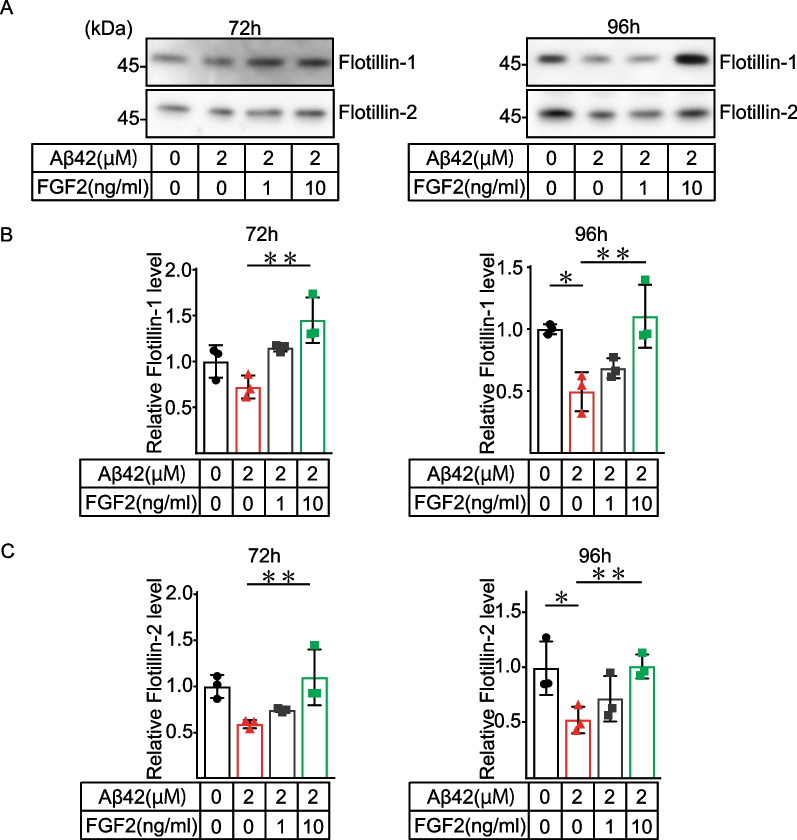
The Aβ42 treatment-induced decrease in the secretion of flotillin on the blood side was restored by the addition of FGF-2. **A** Protein levels were determined by western blotting and quantified by densitometry. **B**, **C** Statistical significance was calculated using one-way ANOVA and the Tukey test (*p < 0.05). Data are shown as the mean ± SD (*n* = 3)

We next investigated the role of FGF-2 in flotillin secretion in the in vitro BBB model. To examine whether the reduced level of FGF-2 in the brain side was responsible for the decreased level of flotillin secretion from iBMECs in the blood side, Aβ42 was added into the brain side in the presence or absence of FGF-2 at concentrations of 1 and 10 ng, and the level of flotillin in the blood side was determined (Fig. 4A). Consistent with the results shown in Fig. 2, Aβ42 treatment of the brain side decreased the secretion levels of flotillin-1 and -2 in the blood side 96 h after treatment (Fig. 4B, C). Of note, this decrease in flotillin secretion was restored in a dose-dependent manner by the administration of recombinant FGF-2 into the brain side. (Fig. 4B, C). Thus, it is likely that the changes in flotillin secretion due to Aβ42 treatment were mediated by FGF-2 from astrocytes. These findings indicated that the decrease in flotillin in the blood-side CM was mediated by decreased FGF-2 production and secretion in astrocytes under the influence of Aβ42.

## Discussion

In this study using the in vitro BBB model, we revealed for the first time the mechanism by which Aβ in the brain affects the blood flotillin level in an endothelial cell-mediated manner. Our findings are as follows. (1) In the in vitro BBB model in which iBMECs were plated on the upper compartment (blood side) and astrocytes were plated on the lower compartment (brain side), Aβ42 treatment of the brain side significantly reduced the level of flotillin released into the blood side. (2) The FGF-2 level in the brain side was significantly reduced by Aβ42 treatment of the brain side. (3) The reduced flotillin release into the blood side induced by Aβ treatment of the brain side was restored in a dose-dependent manner by the addition of FGF-2 into the brain side.

These results showed that the addition of Aβ into the brain side reduced the levels of both cellular FGF-2 in astrocytes and secreted FGF-2 from astrocytes, which may regulate iBMECs to release less flotillin into the blood side. This notion is supported by the fact that Aβ treatment of the brain side of the cultures without astrocytes had no effect on flotillin release from iBMECs into the blood side (Additional file 1: Fig. S1A, B). These results indicated that the effect of Aβ in the brain side on the release of flotillin in the blood side was not a direct effect of Aβ on iBMECs, but an indirect effect via astrocytes. Therefore, it seems reasonable to assume that the reduced level of FGF-2 in the brain side reduced FGF-2-mediated signal transduction via the FGF-2 receptor of iBMECs. This notion is supported by our results shown in Fig. 3D that Aβ treatment suppressed the phosphorylation of FGFR1 in iBMECs (Fig. 3D, E). In line with our findings, previous studies have shown that exogenous basic fibroblast growth factor (FGF-2) treatment preserved the integrity of the BBB, and that secondary brain injury was attenuated via FGFR-induced activation of the PI3K-Akt signaling pathway in mice [13, 14]. In addition, there have been reports that the FGF-2/FGFR1 pathway is involved in vascular remodeling, astrocyte inflammation, and extracellular vesicle (EV) release [15, 16].

The potential usefulness of EVs and exosomes for the diagnosis of AD has been reported [17, 18], and our findings indicated a new mechanism by which high levels of Aβ in the brain may reduce the levels of molecules related to EVs and exosomes, such as flotillin, in blood, providing a rationale for their use as biomarkers for AD. Notably, our finding that Aβ treatment on the brain side reduced flotillin release from iBMECs on the blood side (Fig. 2) indicated a novel mechanism by which brain Aβ may exert its effects on blood molecule(s). Our data also provide a rationale that supports our previous finding that blood flotillin levels are significantly lower in AD patients who are amyloid-PET-positive than in patients with non-AD and vascular dementia [6]. Thus, these results further strengthen the possibility that flotillin may be useful as a diagnostic marker for AD.

The BBB comprises pericytes, astrocytes, and microglia that cooperate to maintain neural circuits, synaptic transmission, synaptic remodeling, angiogenesis, and neurogenesis through cross-talk between these cells [11, 12].

We have shown in Additional file 1: Fig. S4 that co-culture of iBMECs and astrocytes increase FGF-2 levels in astrocytes. These results suggest that iBMECs release some factors to increase FGF-2 levels in astrocytes. Although, the in vitro BBB model used in the present study consisted only of iBMECs and astrocytes, this established co-culture system was used because the iBMECs differentiated from human iPS cells form tight junctions that provide high trans-endothelial electrical resistance (TEER) values (over 1500 Ωm^2^; see Fig. 1C), and the values are much higher than those obtained with other models using rodent cells [11, 12]. Nonetheless, the findings of this study should be confirmed in a BBB model using three types of cells (pericytes, astrocytes, and microglia) in the future.

Astrocytes play an important role in the formation and maintenance of the BBB. During angiogenesis in the central nervous system, astrocytes extend polar endfeet to the luminal side of cerebral blood vessels, and help induce mature cerebral vessels by expressing transporters and anti-permeability proteins, and secreting growth factors [10]. In addition, astrocytes have also been shown to secrete a number of anti-inflammatory and tissue-protective mediators that act on numerous cells to regulate inflammatory conditions [10]. FGF has a multifaceted role in the developing and mature central nervous system, and it has been shown to regulate astrocyte morphogenesis, maturation, and function in both healthy and disease states [10]. In this study, we found that Aβ treatment significantly reduced the level of cellular and secreted FGF-2 in cultured astrocytes (Fig. 3). Previous studies have shown that FGF-2 is one of the most plausible astrocytic factors that induce BBB properties [19] and enhance BBB function in endothelial cells by increasing the expression of occludin and ZO-1 [20]. Consistent with these reports, our findings showed that FGF-2 secreted from astrocytes had an effect on iBMECs, i.e., the FGF-2 at the brain side regulated the secretion of flotillin from iBMECs in the blood side. Based on the findings from a number of recent studies, it was revealed that the relationship between Aβ and BBB can be explained by a “two-hit” theory, i.e., a direct negative effect of cerebrovascular flow reductions and BBB breakdown, leading to neuronal dysfunction and Aβ accumulation (hit 1), and Aβ-mediated BBB dysfunction (hit 2) [21]. Our findings from the present study partly support this idea of brain Aβ-mediated dysregulation of BBB function in terms of the regulation of flotillin release in the blood side.

Based on our data, it is possible that flotillin release from iBMECs in the blood side is regulated by astrocyte-derived FGF-2 in the brain side. Aβ42 attenuated the FGF-2 release from astrocytes, and may therefore lead to reduced flotillin release from iBMECs. Although Aβ affects BBB function by reducing FGF-2 secretion, we did not observe any differences in the TEER values with Aβ treatment (Fig. 1C). One possible explanation is that the decrease in FGF-2 secretion from astrocytes due to Aβ treatment was insufficient to change the TEER values in this BBB model.

In summary, we have identified a novel molecular mechanism by which Aβ in the brain decreases blood flotillin levels, i.e., brain Aβ has an indirect effect on iBMECs to modulate flotillin release from iBMECs. To date, it has been thought that altered metabolites in the brain exit the brain via intramural periarterial drainage or the glymphatic system to blood [22, 23]. Therefore, the pathway revealed in the present study is a novel pathway that differs from the ones that have been hypothesized in the past.

## Supplementary Information

**Additional file 1: Figure S1.** Aβ42 treatment to the brain side did not affect flotillin secretion from iBMECs into the blood side, when iBMECs were cultured without astrocyte (mono-cultured). (A) Flotillin-1, -2 levels were determined by Western blotting and quantified by densitometry. (B) Statistical significance was calculated using the unpaired Student’s *t*-test (ns, not significant). Data are represented as the mean ± SD (*n* = 3).

**Additional file 1: Figure S2.** Aβ42 treatment did not affect the cellular FGF-2 level in iBMECs. Mono-cultured BBB models were treated with or without Aβ42 to the brain side for 96 h. (A) Cellular FGF-2 levels were determined by Western blotting and quantified by densitometry. (B) Statistical significance was calculated using the unpaired Student’s *t*-test (ns, not significant). Data are represented as the mean ± SD (*n* = 3).

**Additional file 1: Figure S3.** Aβ42 treatment tended to but not significantly decrease the cellular FGF-2 level in astrocytes. (A) Astrocyte were treated with or without Aβ42 for 96 h, and cellular FGF-2 levels were determined by Western blotting and quantified by densitometry. (B) Statistical significance was calculated using the one-way ANOVA and Tukey test (*p < 0.05). Data are represented as the mean ± SD (*n* = 3).

**Additional file 1: Figure S4.** Co-culture of iBMECs and astrocytes significantly increased FGF-2 levels in astrocytes. (A) iBMEC and astrocyte were cultured for 96 h, and cellular FGF-2 levels were determined by Western blotting and quantified by densitometry. (B) Statistical significance was calculated using the unpaired Student’s *t*-test (*p < 0.05). Data are represented as the mean ± SD (*n* = 3).

## Data Availability

Data sharing not applicable to this article as no datasets were generated or analyzed during the current study.

## Abbreviations

AD: Alzheimer’s disease
Aβ: Amyloid b-protein
BBB: Blood–brain barrier
FGF-2: Basic fibroblast growth factor
iBMECs: Microvascular endothelial cells obtained from human induced pluripotent stem cells
PET: Positron-emission tomography
CM: Conditioned medium
ELISA: Enzyme-linked immunosorbent assay
TEER: Trans-endothelial electrical resistance
