# Supplementary figures and images for: Aβ42 treatment of the brain side reduced the level of flotillin from endothelial cells on the blood side via FGF-2 signaling in a blood–brain barrier model
Source: Mol Brain. 2023 Jan 26;16:15. doi: 10.1186/s13041-023-01005-1 (PMC9878866; doi:10.1186/s13041-023-01005-1)

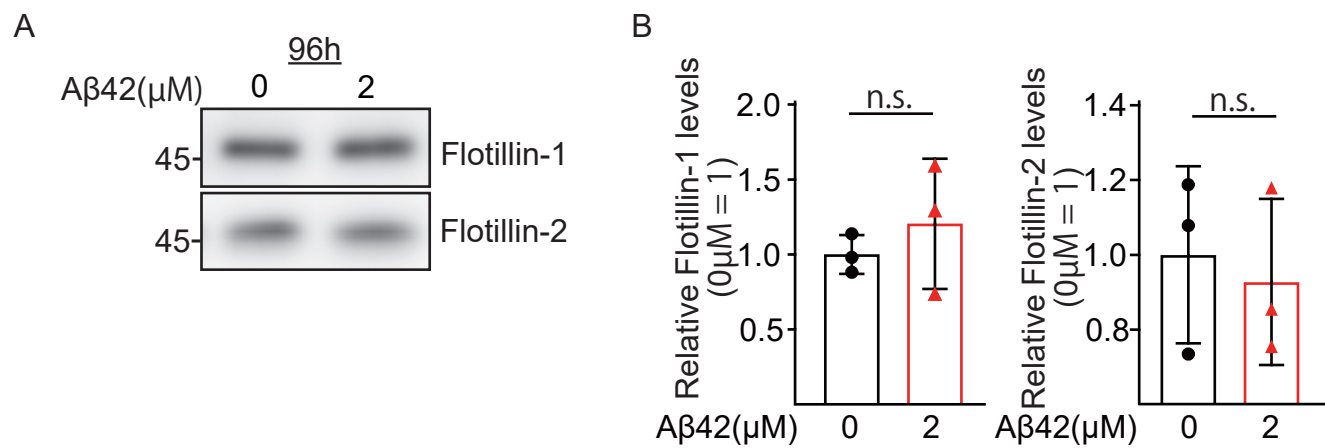

Supplement: Supplementary file 1 — Additional file 1: Figure S1. Aβ42 treatment to the brain side did not affect flotillin secretion from iBMECs into the blood side, when iBMECs were cultured without astrocyte (mono-cultured). (A) Flotillin-1, -2 levels were determined by Western blotting and quantified by densitometry. (B) Statistical significance was calculated using the unpaired Student’s t-test (ns, not significant). Data are represented as the mean ± SD (n = 3). [file 13041_2023_1005_MOESM1_ESM.pdf]

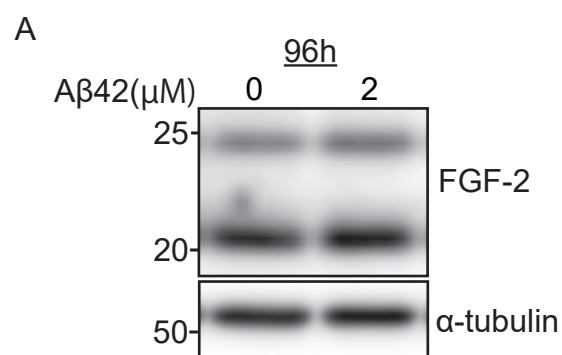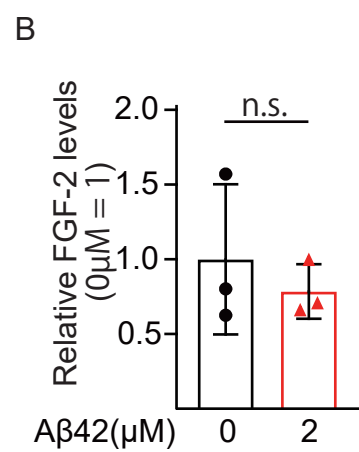

Supplement: Supplementary file 2 — Additional file 1: Figure S2. Aβ42 treatment did not affect the cellular FGF-2 level in iBMECs. Mono-cultured BBB models were treated with or without Aβ42 to the brain side for 96 h. (A) Cellular FGF-2 levels were determined by Western blotting and quantified by densitometry. (B) Statistical significance was calculated using the unpaired Student’s t-test (ns, not significant). Data are represented as the mean ± SD (n = 3). [file 13041_2023_1005_MOESM2_ESM.pdf]

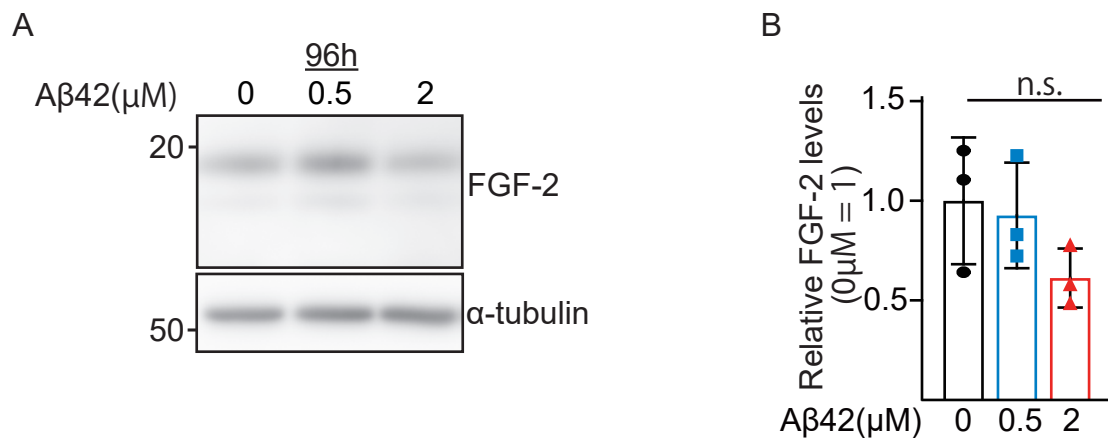

Supplement: Supplementary file 3 — Additional file 1: Figure S3. Aβ42 treatment tended to but not significantly decrease the cellular FGF-2 level in astrocytes. (A) Astrocyte were treated with or without Aβ42 for 96 h, and cellular FGF-2 levels were determined by Western blotting and quantified by densitometry. (B) Statistical significance was calculated using the one-way ANOVA and Tukey test (*p < 0.05). Data are represented as the mean ± SD (n = 3). [file 13041_2023_1005_MOESM3_ESM.pdf]

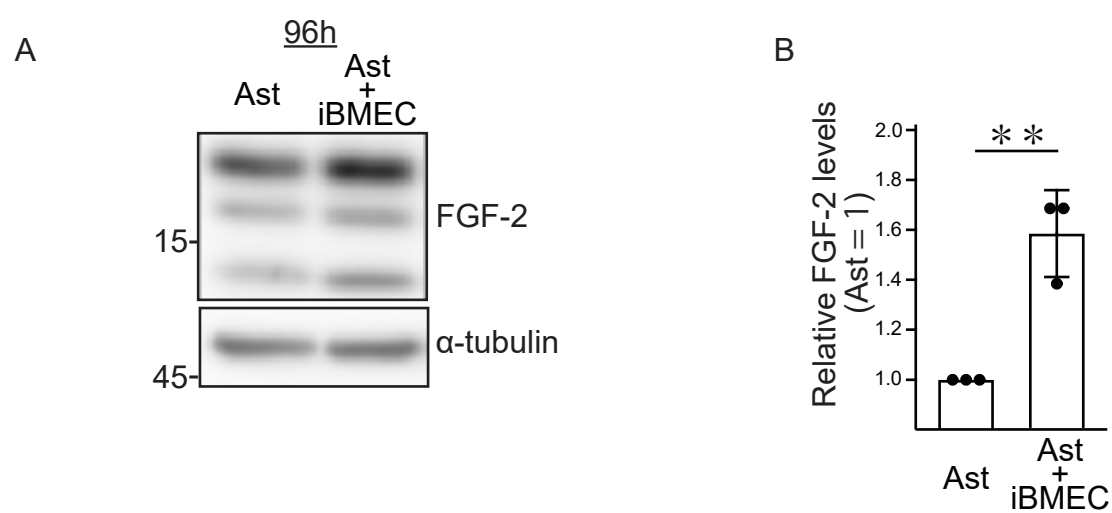

Supplement: Supplementary file 4 — Additional file 1: Figure S4. Co-culture of iBMECs and astrocytes significantly increased FGF-2 levels in astrocytes. (A) iBMEC and astrocyte were cultured for 96 h, and cellular FGF-2 levels were determined by Western blotting and quantified by densitometry. (B) Statistical significance was calculated using the unpaired Student’s t-test (*p < 0.05). Data are represented as the mean ± SD (n = 3). [file 13041_2023_1005_MOESM4_ESM.pdf]
